# Optimization, partial purification, and characterization of a novel high molecular weight alkaline protease produced by *Halobacillus* sp. HAL1 using fish wastes as a substrate

**DOI:** 10.1186/s43141-023-00509-6

**Published:** 2023-05-01

**Authors:** Nayer M. Fahmy, Bahig El-Deeb

**Affiliations:** 1 Marine Microbiology Laboratory, National Institute of Oceanography & Fisheries, Cairo, Egypt; 2 Faculty of Science, Botany and Microbiology Department, Sohag University, Sohag, Egypt

**Keywords:** Halobacillus, Fish processing wastes, Alkaline protease, Saline soil

## Abstract

**Background:**

Hydrolytic enzymes from halophilic microorganisms have a wide range of industrial applications. Herein, we report the isolation of *Halobacillus* sp. HAL1, a moderately halophilic bacterium that produces a novel high molecular weight extracellular alkaline protease when grown in fish processing wastes as a substrate.

**Results:**

Results showed that the isolated strain belonged to the genus *Halobacillus*, and it was designated as *Halobacillus* sp. HAL1 with the GenBank accession number OK001470. The strain secreted an extracellular alkaline protease, and the highest yield was obtained when it was grown in a medium with fish wastes substrate as the sole nutritional source (10 g/L) and incubated at 25 °C under shaking conditions. The enzyme was partially purified by Sephadex G-100 column chromatography. Zymographic analysis showed two casein degrading bands of about 190 and 250 KDa. The optimum enzyme activity was at a temperature of 50 °C at pH 8. The proteolytic activity was enhanced in the presence of metal ions (Ca^2+^, Mg^2+^, and Mn^2+^), surfactants (Tween 80, SDS, and Triton-X100), H_2_O_2_, and EDTA.

**Conclusion:**

Our study indicates that *Haobacillus* sp. HAL1 is a moderately halophilic strain and secrets a novel high molecular wight alkaline protease that is suitable for detergent formulation.

## Background

Microorganisms are a valuable source of enzymes for both industrial and medical uses because of their rapid growth rate and simplicity of manipulation, especially with the advent of recombinant DNA technology and protein engineering [1]. Microbial enzymes have been employed in the catalytic bioprocesses of a variety of industries, including food, agriculture, chemicals, medicine, and energy. Microbial enzymes are preferred over plant and animal enzymes in industry and medicine due to their stability, higher catalytic activity, regular supply, greater yield, and lower cost of recovery from the producing microbes. Furthermore, as compared to traditional catalytic methods, microbial enzymes perform well under a wide range of chemical and physical conditions, are more efficient, produce high-quality products, and are less harmful to the environment [2, 3]. The development of novel, economically competitive, and sustainable production processes necessitates the rapid discovery of novel enzymes with unique properties [4].

Due to their highly flexible metabolism, extremophilic bacteria have adapted to survive in extreme conditions (e.g., high/low temperature, pH, salinity, and pressure) and are potential sources of catalytically stable enzymes (proteases, amylases, lipases…etc.) that could work under harsh industrial conditions [5–7] and therefore, are attractive for different industries, especially those including high salt concentrations, such as textile, fermented food, pharmaceuticals, cosmetics, and leather industries [8–10].

Proteases are widely used in the food, detergent, and pharmaceutical industries. They represent about 60% of the industrial enzyme market, with increasing global demand during 2014–2019 at a compound annual growth rate (CAGR) of 5.3% and is expected to increase significantly as they become more widely applied in bioremediation and the leather processing industries [3, 11]. Microbial proteases account for about two-thirds of commercial proteases because they have the characteristics required for industrial applications (e.g., less time consumption, high yield, cost-effectiveness, less space requirement, and genetic manipulation) compared to plant and animal proteases [12, 13]. Microbial proteases are classified into acidic and alkaline proteases based on their pH range of activity. Acidic proteases are active at acidic pH, while alkaline proteases are active at alkaline pH. Among microorganisms, bacteria are the primary source of alkaline proteases, with the *Bacillus* genus being the most prolific producer and the most commercially exploited microbes [13].

The high cost of substrates is the most important factor limiting the production of microbial enzymes for industrial applications; thus, using low-cost substrates is important from a commercial standpoint [14, 15]. The search for low-cost substrates suitable for microbial enzyme production is critical [15–17]. Fish processing waste (FPW) is a low-cost nutritional substrate that is suitable for the growth of enzyme-producing microorganisms and can be used to produce enzymes. Several studies reported the use of FPW to produce microbial enzymes [18].

Sabkhas or “salt falts” are saline environments that are periodically inundated with water, and evaporites are formed due to capillary evaporation [19]. Microorganisms that are halophilic or halotolerant inhabit these environments [20]. Despite the prevalence of sabkha environments along the Egyptian Red Sea coast, little is known about the microorganisms inhabiting these environments. The sabkha of wadi abu-Shaar, located north of Hurghada City on the Egyptian Red Sea coast, is one of these environments that has not been extensively studied. The current study focuses on the optimization of production, partial purification, and characterization of a high molecular weight alkaline protease produced by *Halobacillus* sp. HAL1, which was recently isolated from the saline soil of wadi abu-Shaar, north of Hurghada City on Egypt’s Red Sea coast.

## Methods

### Study area

**Fig. 1:**
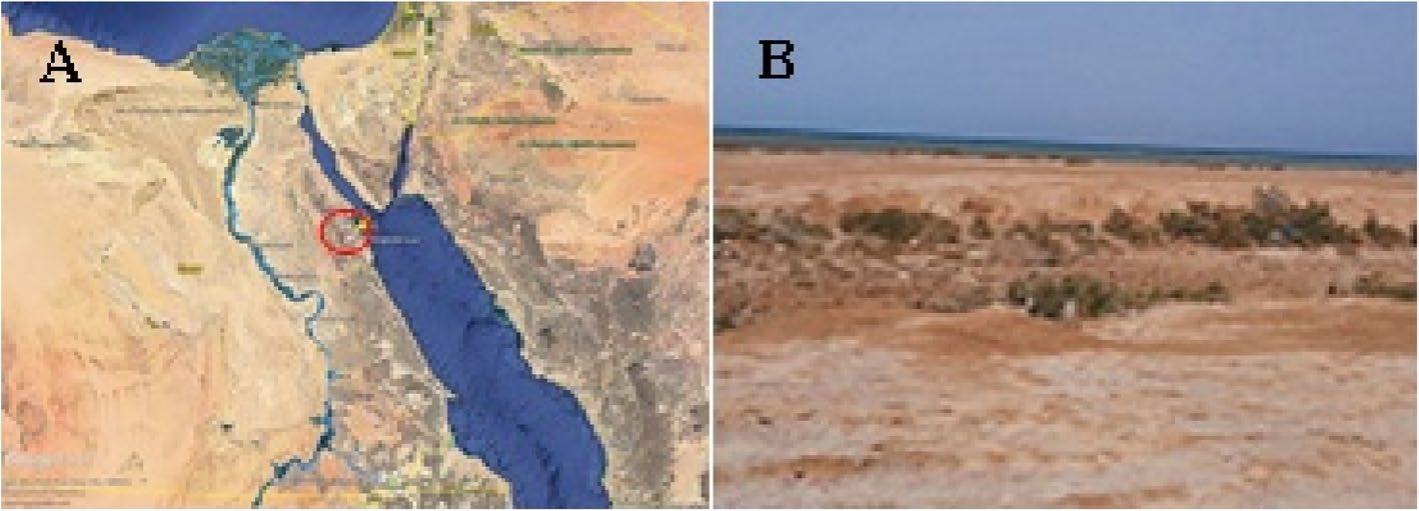
**a** Map of Egypt showing the location of Wadi Abu-Shaar (red circle). **b** A photo of saline soil in the Wadi Abu-Shaar area (photo taken by authors)

Wadi Abu-Shaar is about 10 km north of Hurghada City, between the latitudes of 27°18′25′′N and 33°43′15′′E (Fig. 1a). In the backshore area, there are sabkha evaporites and dwarf sand dunes covered in rare plants (Fig. 1b). Five different sediment samples were collected using a sterile scooper from the top 10 cm of saline soil and stored at 4 °C in sterile polyethylene bags. Within a few hours, the samples were transported to the laboratory and processed.

### Isolation of heterotrophic bacteria

For isolation of salt-tolerant heterotrophic bacteria, 1 g of each sediment sample was homogenized in 9 mL of sterilized seawater and serially diluted up to 10^−5^. The serial dilutions of all samples were plated in tryptone soya agar medium prepared using artificial seawater containing 80 gm/L of NaCl. The plates were incubated for 24 h at 37 °C. One plate yielding well isolated colonies from each sample was selected, and one colony from each morphotype was picked and purified by streaking 2–3 times to ensure purity of the isolates. Pure cultures were stored at 4 °C [21]. Six bacterial isolates (HAL1-HAL6) were obtained and screened for alkaline protease production.

### Screening for alkaline protease production

To evaluate the proteolytic activity of the bacterial isolates, Horikoshi-I alkaline medium [22] was used with some modifications. The alkaline agar medium (pH 9) contained (g/L): glucose 10, peptone 5, yeast extract 5, Mg_2_SO_4_. 7H_2_O 0.2, K_2_HPO_4_ 1, NaCl 50, Na_2_CO_3_ 10, and agar 15. Skim milk (10%, v/v) was supplemented to the medium as an indicator for proteolytic activity. Glucose and Na_2_CO_3_ solutions were autoclaved separately, cooled down, and added to the autoclaved medium. Sterile filter paper discs, 5 mm in diameter, were impregnated separately with 30 μL of lag phase cultures of each isolate and put on the alkaline agar medium plates. The plates were incubated for 24 h at 35 °C, and the appearance of clear zone around the colonies was taken as evidence for the production of alkaline protease [23].

### Identification of bacteria

The bacterial isolate (HAL1) with the highest alkaline protease production was identified based on its morphological and biochemical characteristics as described in Bergey’s manual of determinative bacteriology [24]. The isolate was further identified using 16 s rDNA sequence analysis. Genomic DNA was extracted using the Hipura Bacterial DNA Kit (Angen Biotech, China) according to the manufacturer’s instructions. PCR amplification of the 16 s rDNA was carried out using the forward primer: 16F 27 (5′-AGA GTT TGA TCC TGG CTC AG-3′), and the reverse primer: 16R 1525 (5′-AAG GAG GTG ATC CAG CCG CA-3′). The PCR product was purified using the QIA quick gel extraction kit (Qiagen, USA) and sequenced using an automated sequencer (Macrogen, Korea). The identity of the isolate was determined by aligning the obtained sequence with the reference sequences available on the NCBI homepage using the BLAST algorithm (www.ncbi.nlm.nih.gov/blst). Multiple alignments and phylogenetic tree construction were performed using the Neighbor-Joining method using Mega-X software, version 10.1.7 [25].

### Growth conditions

To study the optimal growth conditions, the HAL1 strain was grown in the medium described above, without the addition of agar. The amount of NaCl was investigated in the range of 0–25%. The pH range was 4–10, and the temperature was 10–45 °C. All experiments were carried out in triplicate with shaking at 150 rpm. The bacterial growth was monitored by measuring the absorbance at 600 nm [26].

### Preparation of fish wastes substrate

Fish by-products (viscera and head contents) of *Scarus collana* were obtained from a fish market in Hurghada, Egypt. To obtain fish wastes flour, viscera and head contents were cooked until boiling. The cooked materials were pressed to remove water and oil, dried for 24 h at 80 °C, and grinded [27].

### Production of alkaline protease

The effectiveness of fish waste flour as a substrate for the production of HAL1 protease was tested using the following media:

- YT medium (g/L): Peptone 10, yeast extract 1, K_2_HPO_4_ 1, MgSO_4_. 7 H_2_O 1, MnSO_4_. 7 H_2_O 0.1, glucose 2 [27]; SCG medium (g/L): *Scarus collana* (SC) waste flour 10, K_2_HPO_4_ 1, MgSO_4_. 7 H_2_O 1, MnSO_4_. 7 H_2_O 0.1, glucose 2; SC medium (g/L): SC flour 10. Synthetic seawater containing 90 g/L of NaCl was used to prepare all of the culture media. A volume of 50 mL of each medium was inoculated with 0.1 mL of HAL1 isolate suspension (A600 nm = 0.4) in 250 mL Erlenmeyer flasks, pH 9, and incubated for 24 h at 35 °C with shaking at 150 rpm. To maintain the alkaline condition, each culture was buffered with 50 mM Tris–HCl buffer (pH 9).

### Determination of enzyme activity

Protease activity was measured according to previously described methods by Cupp-Enyard [28] with some modifications. Briefly, the reaction system (1.0 mL) composed of 250 μL of 0.65% casein in 100 mM Tris–HCl buffer (pH 8) and 250 μL of appropriately diluted cultivated supernatant, which was incubated for 30 min at 37 °C. The reaction was terminated by adding 500 μL of trichloroacetic acid (110 mM). Then it was centrifuged for 10 min at 10,000 rpm. Five hundred microliters of Na_2_CO_3_ (500 mM) and 0.3 of appropriately diluted Folin-Ciocalteu reagent were added to 0.2 mL of the supernatant, mixed thoroughly, and incubated for 30 min at 37 °C. To determine the amount of tyrosine liberated from the substrate, the absorbance of all sample replicates and the blank (containing deionized water instead of the enzyme solution). was measured at 660 nm using a JEN-WAY 6800 spectrophotometer. A standard curve was developed using tyrosine (0–16 μg/mL) as a standard substance. The absorbance response (*y*) of tyrosine (*y* = 0.0708*x* + 0.0056, *R*^2^ = 0.9894) and concentrations (0–16 μg/mL) was linear. One unit of enzyme activity was defined as the quantity of protease that liberates 1 μg/mL of tyrosine per min.

### Optimization of alkaline protease production

#### Effect of NaCl concentration

To determine the optimum concentration of NaCl for alkaline protease production by strain HAL1, the strain was grown in SC medium supplemented with different concentrations of NaCl (1–15%) and incubated for 24 h at 30 °C under shaking conditions (150 rpm). All of the experiments were carried out in triplicate and the protease activities were determined.

#### Effect of fish waste substrate concentration

The effect of the concentration of *Scarus collana* waste flour on alkaline protease production was investigated at a range of 5–40 g/L, keeping all other parameters constant.

#### Effect of pH, temperature, and aeration

The influence of initial pH on alkaline protease production by strain HAL1 was investigated by culturing the strain in SC medium with different pH (6-11) while keeping all other parameters constant. Similarly, to investigate the effect of temperature on the alkaline protease bioprocess, the strain was grown under various temperatures (20–40 °C) at the optimum growth parameters. The effect of aeration on protease production was also investigated by growing the strain under static and shaking (150 rpm) conditions [28]. All of the experiments were done in triplicate, and the enzyme activities were assayed as described above.

### Fermentation and partial purification of the enzyme

Strain HAL1 was grown at pH 8 in a protease production medium prepared using artificial sea water and containing SC waste powder (10 g/L) and NaCl (3 g/L), with pH 8. The medium was autoclaved for 20 min at 121 °C, cooled to room temperature, and inoculated with 0.5% (v/v) of a 24-h-old culture of HAL1. The Fermentation process was carried out under shaking conditions (150 rpm) for 36 h at 25 °C. To separate the biomass, the culture was centrifuged at 4 °C for 10 min at 10,000 rpm. The enzyme was precipitated from the supernatant by adding two volumes of chilled acetone. The precipitated protein was dissolved in phosphate buffer (pH 8).

### Gel filtration chromatography

Concentrated enzyme solution was applied onto gel filtration Sephadex G-100 column (ID 0.8 cm × BH 84 cm) previously equilibrated with 0.1 M Tris–HCl buffer pH 8. The column was eluted with 0.1 M Tris–HCl buffer pH 8. The flow rate was maintained at 20 mL/h and fractions of 3 mL were collected and dialyzed against the same buffer. The content of protein and the activity of the enzyme were determined for each fraction, and the fraction with the highest activity per mg of protein was chosen for enzyme characterization and zymography development.

### Zymographic analysis

Zymogram analysis was performed according to Garcia-Carreno et al. [29] with some modifications. Briefly, samples were separated on a 10% resolving gel supplemented with 0.1% casein as a copolymerized substrate. After separation, the gel was rinsed with distillated water and agitated in phosphate buffer (pH 8.3) containing Triton X-100 (2.5%) for 60 min. Subsequently, the gel was incubated in phosphate buffer (pH 8.3) at 50 °C for 12 h. Finally, the gel was stained with Coomassie brilliant blue R-250. The presence of alkaline protease activity was indicated by the appearance of a clear zone on a dark blue background.

### Characterization of the enzyme

#### Effect of pH, temperature, and salt concentration

Various buffers (50 mM) with different pH values (3-11) were used to test the effect of pH on protease activity: citrate buffer (pH 3–6), phosphate buffer (pH 6–8), Tris–HCl (pH 8–9), and glycine–NaOH (pH 9–11). Fifty microliters of the enzyme was added to 200 µL of the appropriate buffer and mixed with 250 µL of 0.65% casein dissolved in distillated water, and the assay was carried out as described above. To determine the optimum temperature for protease activity, the assay was carried out at various temperatures (5–80 °C). The assay was performed in the presence of 0–4 M NaCl or 0–3 M KCl at the optimal pH and temperature to determine the effect of salt concentration on protease activity [30].

#### Effect of organic solvents

The effect of various organic solvents, including methanol, ethanol, xylene, acetone, hexane, benzene, propanol, and butanol, on protease activity was investigated. The partially purified enzyme was pre-incubated with each organic solvent at a 25% final concentration in 50 mM tris–HCl buffer (pH 8) for 10 min at 37 °C, and the assay was performed as described above. The activity of the enzyme was assumed to be 100% in the absence of organic solvent [31].

#### Effect of surfactants, EDTA, and H_2_O_2_

The effect of surfactants (SDS, Tween 80, and Triton X-100) and H_2_O_2,_ on protease activity was determined by pre-incubating the enzyme with different concentrations (1, 5, and 10%) of the respective agent for 10 min. The effect of different concentrations of EDTA (1, 5, and 10 mM) on protease activity was studied by pre-incubating the enzyme with EDTA for 10 min. Protease activity was carried out for 1 h at the optimum pH and temperature and assayed as mentioned above. The enzyme activity without any surfactant, H_2_O_2_, or EDTA was regarded as 100% [31].

### Statistical analysis

All experiments and assays were carried out in triplicates. Means and standard deviations were calculated by Microsoft Excel software.

## Results

### Isolation of bacteria and screening for alkaline protease production

**Fig. 2:**
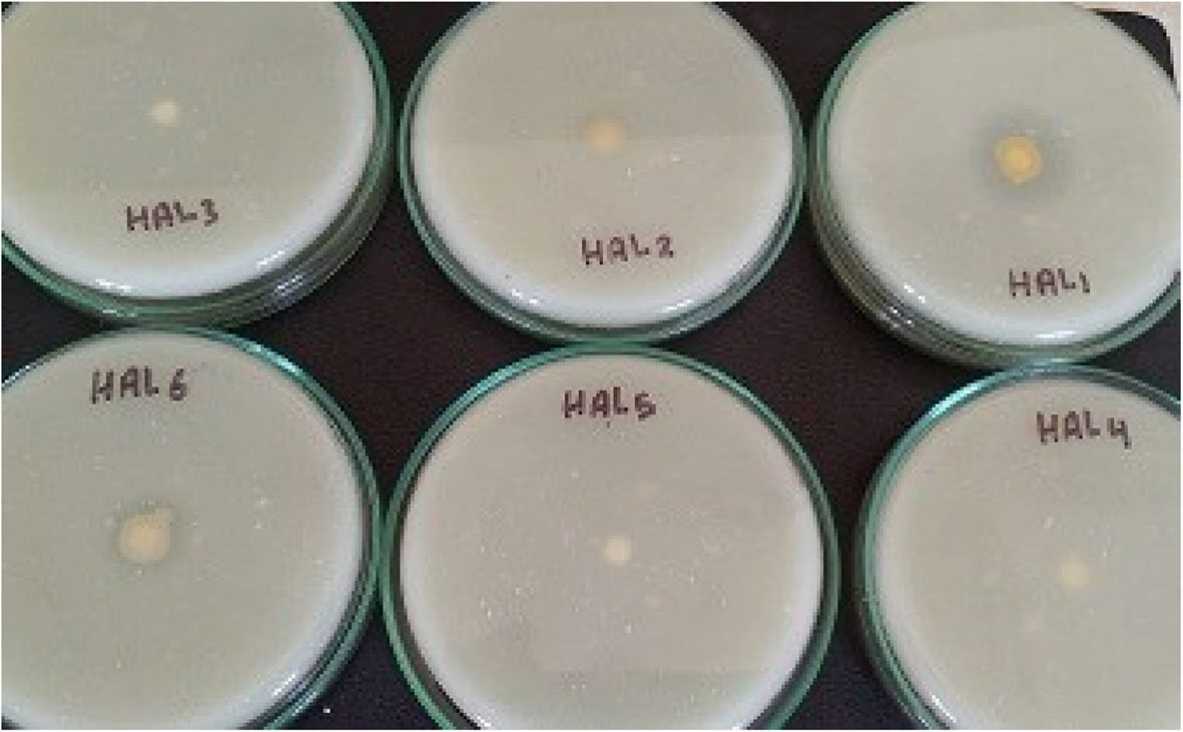
Screening of bacterial isolates for protease production. Isolates were grown on alkaline agar medium supplemented with skimmed milk and 9% NaCl, incubated for 24 h at 37 °C. The proteolytic activity is shown by the hydrolysis of skimmed milk

In the present study, the isolation of salt-tolerant heterotrophic bacteria from saline sediment yielded six bacterial isolates (HAL1-HAL6). Using alkaline agar medium supplemented with skimmed milk and 9% NaCl, all of the isolates were screened for the production of alkaline protease. Due to the highest proteolytic activity of the HAL1 isolate in comparison with the other isolates, it was chosen for further characterization and alkaline protease production (Fig. 2).

### Morphological and biochemical characterization

Morphological and biochemical characterization of the HAL1 isolate revealed that colonies were yellow orange, smooth, circular, slightly raised, and approx. 2 mm in diameter after incubation for 2 days at 30 °C on nutrient agar supplemented with 10% NaCl (w/v). The pigment was not soluble in water and non-diffusible. HAL1 grew in a wide range of NaCl concentrations (1–21%, w/v) and optimally at 9% (w/v) NaCl. Furthermore, the isolate was aerobic, spore-forming, and grew optimally at 35 °C. The morphological and biochemical characteristics of the HAL1 isolate and the closely related bacterial species are depicted in Table 1.

**Table 1:** Morphological and biochemical characteristics of *Halobacillus* sp. HAL1 in comparison with related species

Characteristics	1	2	3
Morphology	Rods, single, or in chain	Rods, single, or in chain	Rod
Pigmentation	Yellow orange	white	Orange
Spore shape	S/E	S/E	E/S
Spore position	C/ST	C/ST	C/ST
Motility	−	−	+
Gram	+	+	+
NaCl concentration for growth (%, w/v)			
Range	1–21	1–24	0.5–30
Optimum	9	10	10
Temperature range for growth (°C)	25–40	10–49	10–44
pH range for growth	7–10	6–9.6	6–9.5
Hydrolysis of			
Gelatin	+	+	+
Casein	+	+	−
Starch	+	+	−
Urea	−	−	−
Acid from			
D-galactose	−	−	+
glucose	+	+	+
Maltose	+	+	+
Xylose	−	−	−

Strains: *1*, *Halobacillus* sp. HAL1; *2*, *Halobacillus karajensis* [32];* 3*, *Halobacillus trueperi* DSM 10404 T [33]

### Molecular identification

**Fig. 3:**
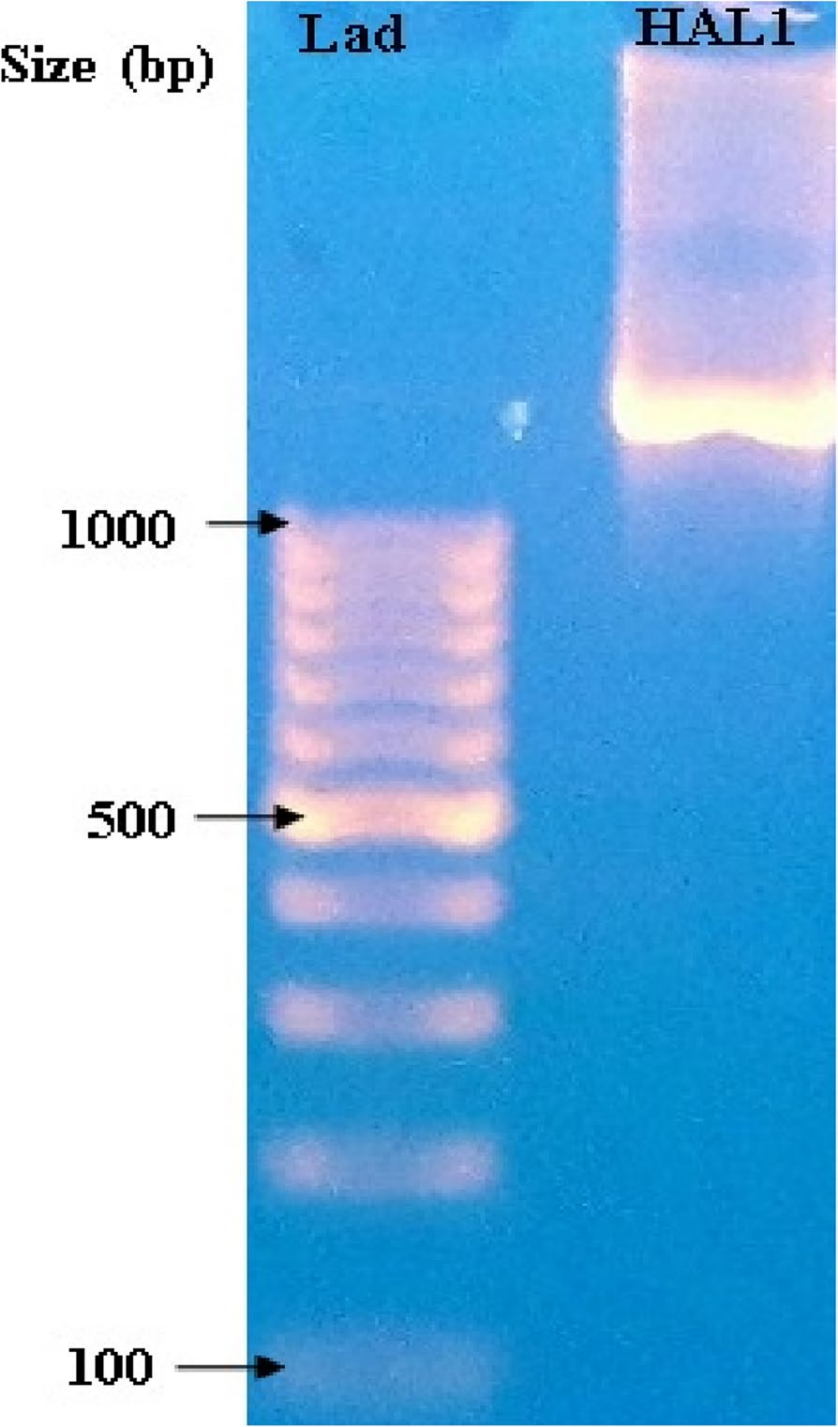
PCR amplified product of 16S rDNA of the HAL1 strain (Lad: molecular size marker 100–1000 bp; HAL1: PCR product of HAL1 isolate)

**Fig. 4:**
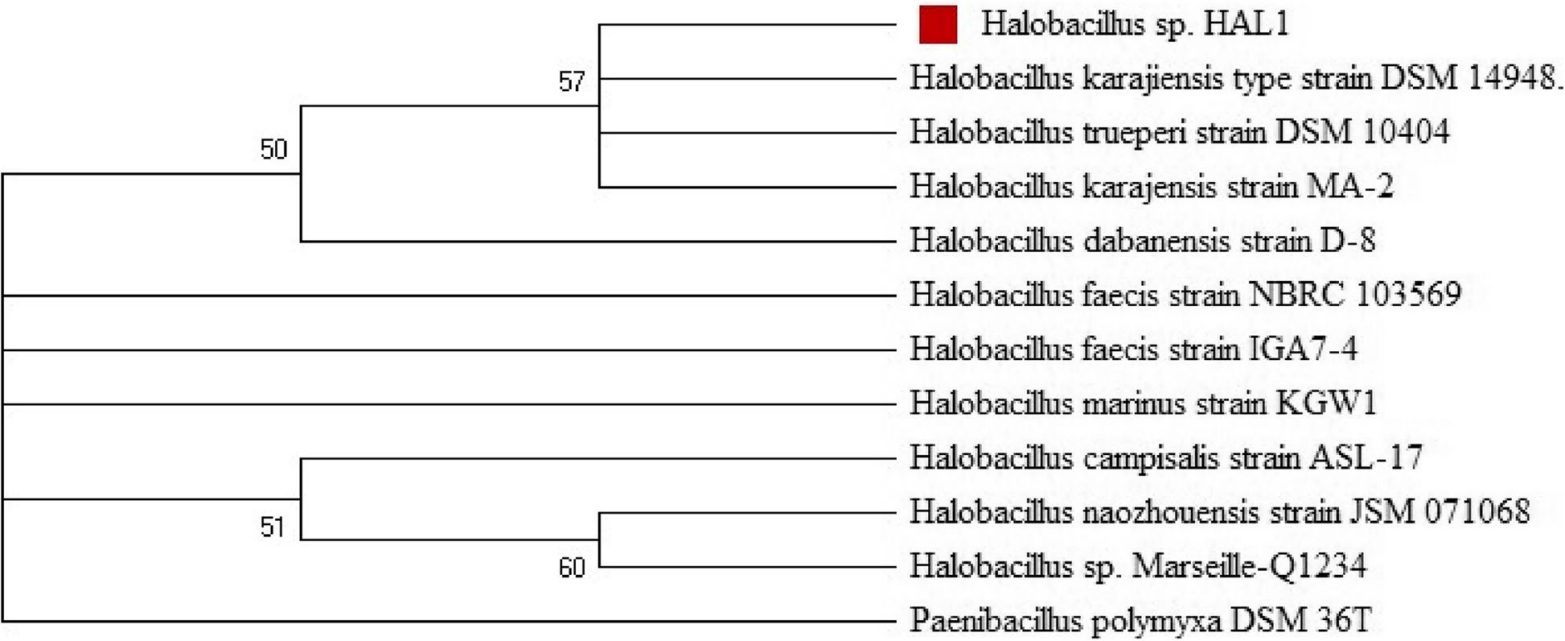
Neighbor-joining phylogenetic tree of *Halobacillus* sp. HAL1 and the related *Halobacillus* species. Numbers at nodes are bootstrap percentages based on 1000 resamplings. Only values above 50 are given

The 16 s rRNA gene was amplified using PCR to determine the taxonomic position of the strain HAL1. The 1226 pb amplified product (Fig. 3) was sequenced and compared to the sequences in the NCBI nucleotide database using the BLAST algorithm (http://blast.ncbi.nlm.nih.gov/). The BLAST search revealed that the strain belongs to the genus *Halobacillus* and exhibited high similarity to many species of the genus: *H. trueperi* strain DSM 10404 (GenBank accession no. NR_025459, 99.87% similarity), *H. karajiensis* strain DSM 14948 (GenBank accession no. AJ486874, 99.74% similarity), *H. dabanensis* strain D-8 (GenBank accession no. NR_042860, 99.74% similarity), and *H. faecis* strain NBRC 103569 (GenBank accession no. NR_114247, 99.61% similarity). The sequence was deposited in the GenBank as *Halobacillus* sp*.* strain HAL1 with an accession number of OK001470. Figure 4 shows the phylogenetic relationship with the related *Halobacillus* spp. *Paenibacillus polymyxa* strain DSM 36^ T^ was used as an outgroup to root the tree.

### Production of alkaline protease

**Fig. 5:**
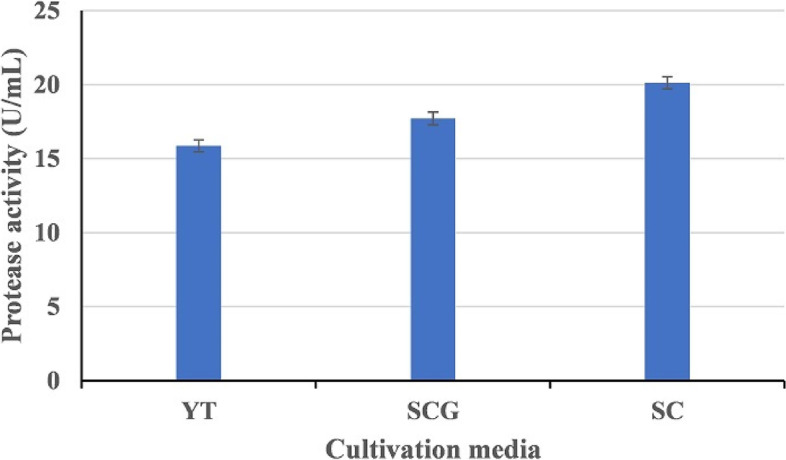
Effect of cultivation media on the production of alkaline protease by strain HAL1 after incubation for 24 h at 37 °C under shaking conditions (150 rpm). Each value is a mean of three cultures, and standard deviations are presented as error bars (*n* = 3). YT medium: containing peptone, yeast extract, glucose, and salts; SCG medium: containing fish waste substrate, salts, and glucose; SC medium: containing only fish waste substrate

To test the suitability of *S. collana* waste substrate for alkaline protease production by strain HAL1, three culture media were used, all of which were made with synthetic seawater: YT medium, which contained commercial substrates, and two other media (SCG and SC), which were made with fish waste substrate flour. The results revealed that the SC medium, which contained only fish waste flour, supported higher levels of protease production (20.2 ± 0.40 U/mL) than the other media (Fig. 5). Protease activity was 17.71 ± 0.41 U/mL in the SCG medium, which contained both fish waste flour and glucose, and 14.87 ± 0.41 U/mL in the YT medium, which contained yeast extract and peptone. Based on these findings, SC medium prepared using synthetic seawater and containing only *S. collana* waste flour was chosen for optimization of the alkaline protease bioprocess by the HAL1 strain.

### Optimization of protease production

#### Effect of NaCl concentration

**Fig. 6:**
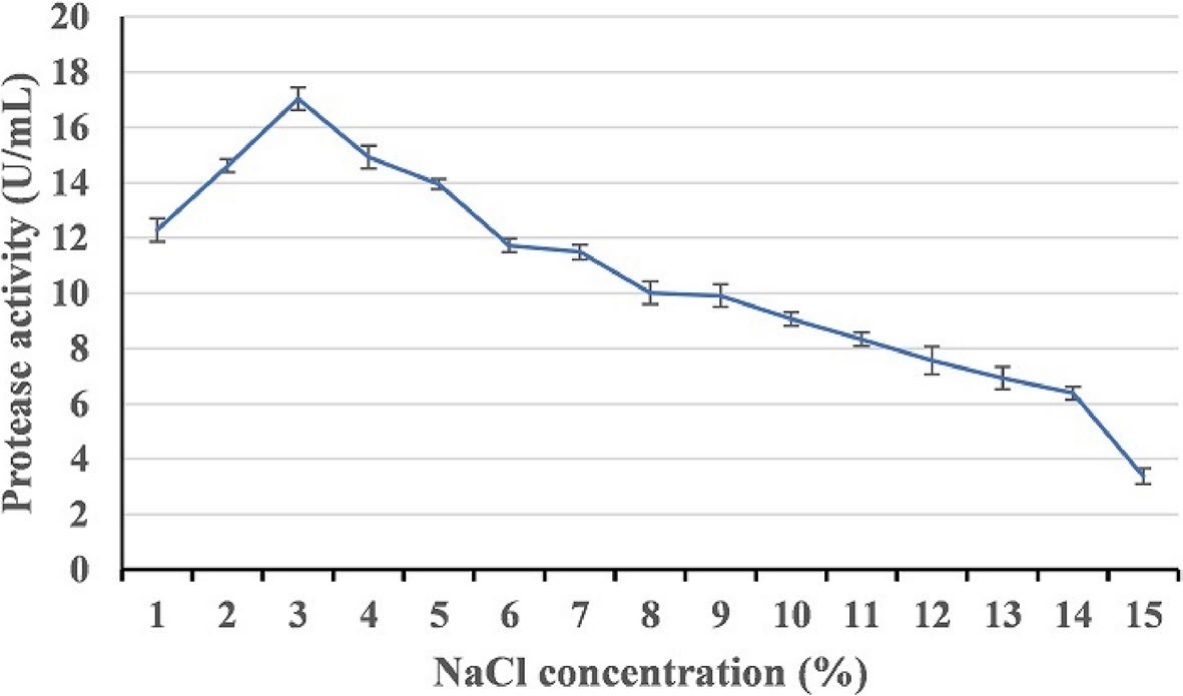
Effect of sodium chloride concentration on the production of alkaline proteases by strain HAL1 after incubation for 24 h at 37 °C under shaking conditions (150 rpm). Each value is a mean of three cultures and standard deviations are presented as error bars (*n* = 3)

The effect of sodium chloride concentration (1–15%) on the production of alkaline protease by strain HAL1 was studied. The strain produced the highest amount of protease in the medium containing 3% NaCl. However, a further increase in NaCl concentration caused a drastic decrease in alkaline protease production (Fig. 6).

#### Effect of substrate concentration

**Fig. 7:**
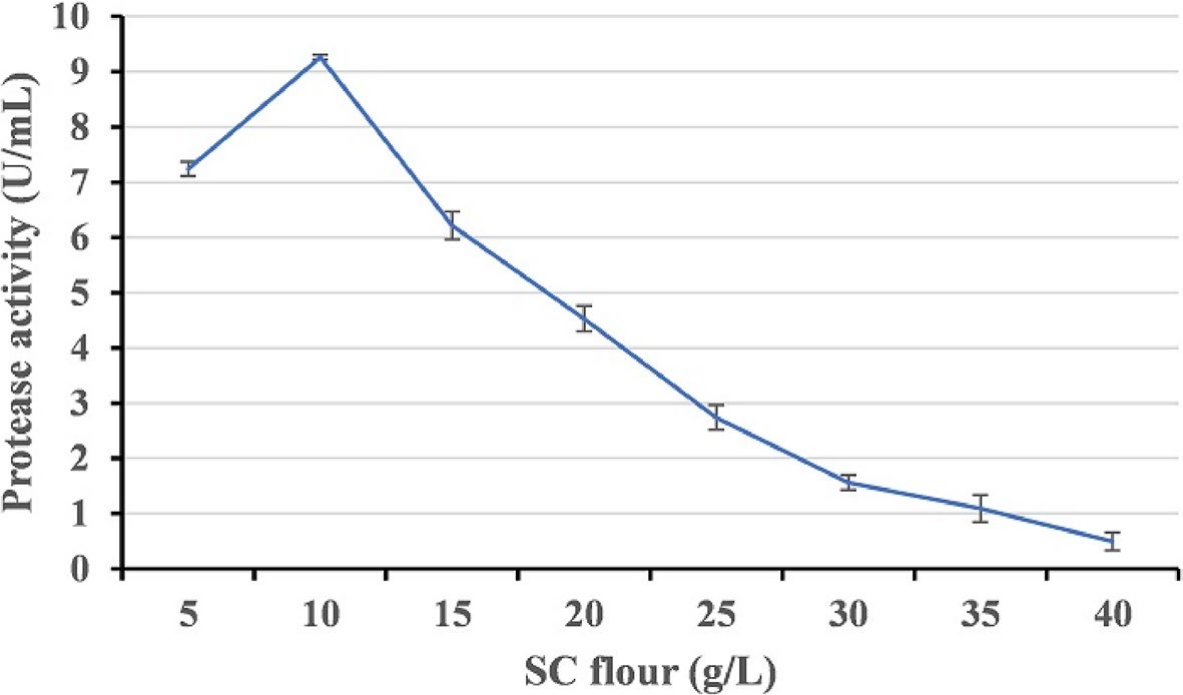
Effect of SC flour concentration on the production of alkaline proteases by strain HAL1 after incubation for 24 h at 37 °C under shaking conditions (150 rpm). Each value is a mean of three cultures, and standard deviations are presented as error bars (*n* = 3)

The effect of varying concentrations of *S. collana* waste flour on the production of alkaline protease was investigated at a range of 5–40 g/L. The results showed that at a concentration of 10 g/L, high alkaline protease production was achieved. However, a further increase in the concentration of fish waste flour caused a decrease in protease production (Fig. 7).

#### Effect of pH, temperature, and aeration

**Fig. 8:**
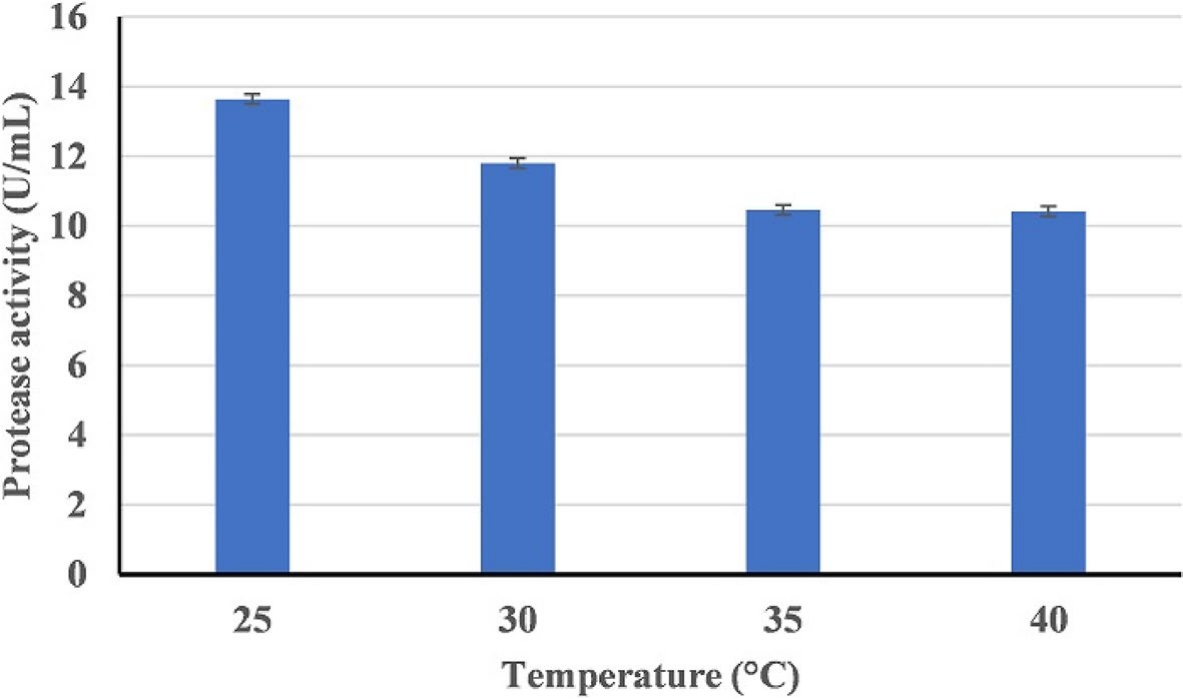
Effect of temperature on the production of alkaline proteases by strain HAL1 after incubation for 24 h under shaking conditions (150 rpm). Each value is a mean of three cultures, and standard deviations are presented as error bars (*n* = 3)

**Fig. 9:**
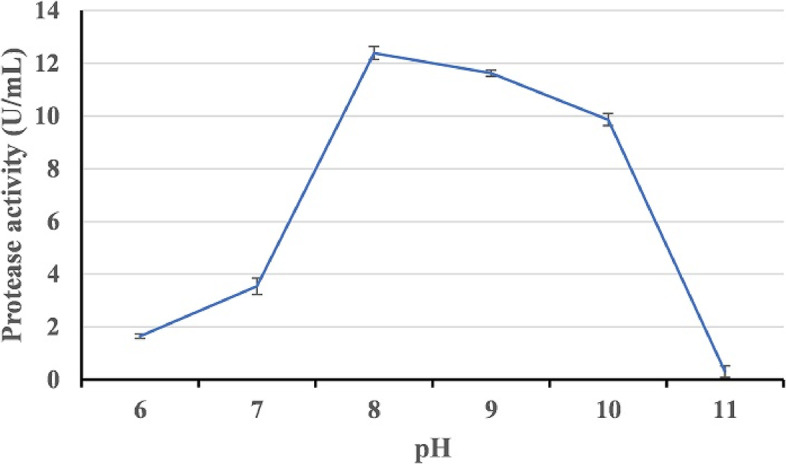
Effect of pH on the production of alkaline proteases by strain HAL1 after incubation for 24 h at 25 °C under shaking conditions (150 rpm). Each value is a mean of three cultures, and standard deviations are presented as error bars (*n* = 3)

**Fig. 10:**
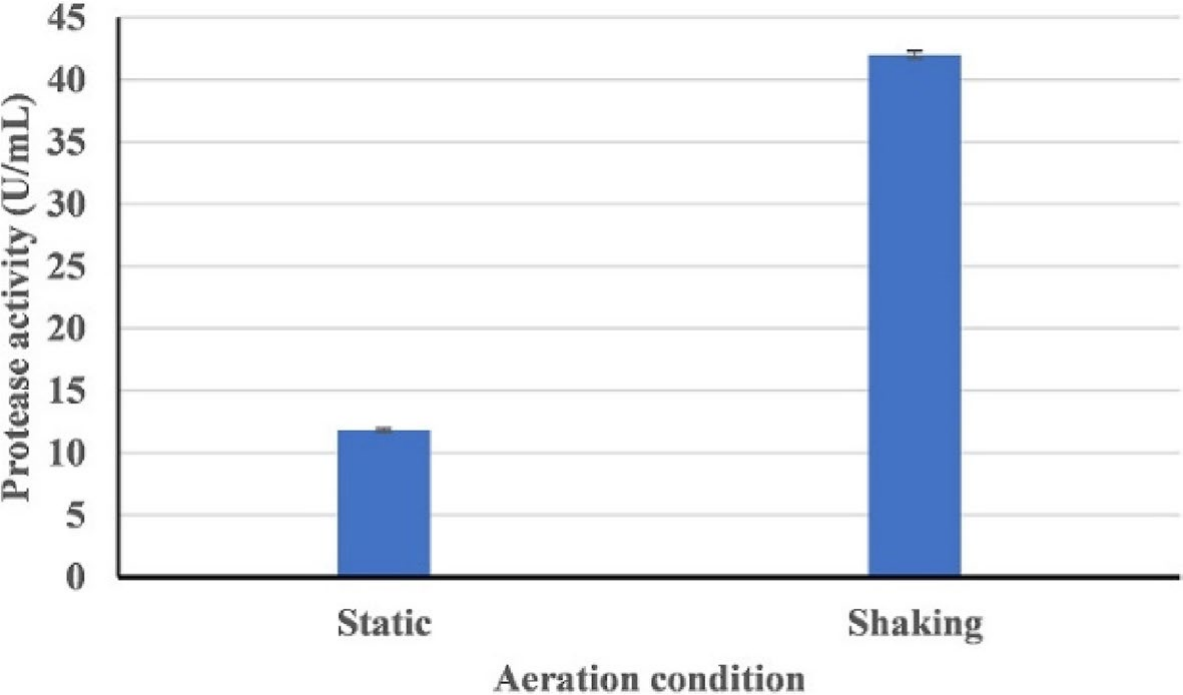
Effect of aeration on the production of alkaline proteases by strain HAL1 after incubation for 24 h at 25 °C under static and shaking conditions (150 rpm). Each value is a mean of three cultures, and standard deviations are presented as error bars (*n* = 3)

Figure 8 shows the effect of the incubation temperature (25–40 °C) on HAL1 protease production. The optimum temperature for the production of alkaline protease was found to be 25 °C (13.63 U/mL). With increasing growth temperature, there was a slight decrease in enzyme production, with the enzyme yield dropping to 10.41 U/mL at 40 °C. With regard to the pH effect, strain HAL1 was able to produce alkaline protease over a wide pH range (6-10) with maximum enzyme production at pH 8 (Fig. 9). Furthermore, aeration of the culture had a significant impact on enzyme production. The enzyme yield of the culture incubated under shaking conditions (150 rpm) was about 4 folds compared to static conditions, 40 and 11.8 U/mL, respectively (Fig. 10).

### Partial purification and characterization of the enzyme

The partial purification of the protease enzyme was carried out with Sephadex G-100 column chromatography. The activity of the different fraction per mg of protein was determined. The fraction with the highest activity of protease was chosen for further characterization.

### Zymogram analysis

**Fig. 11:**
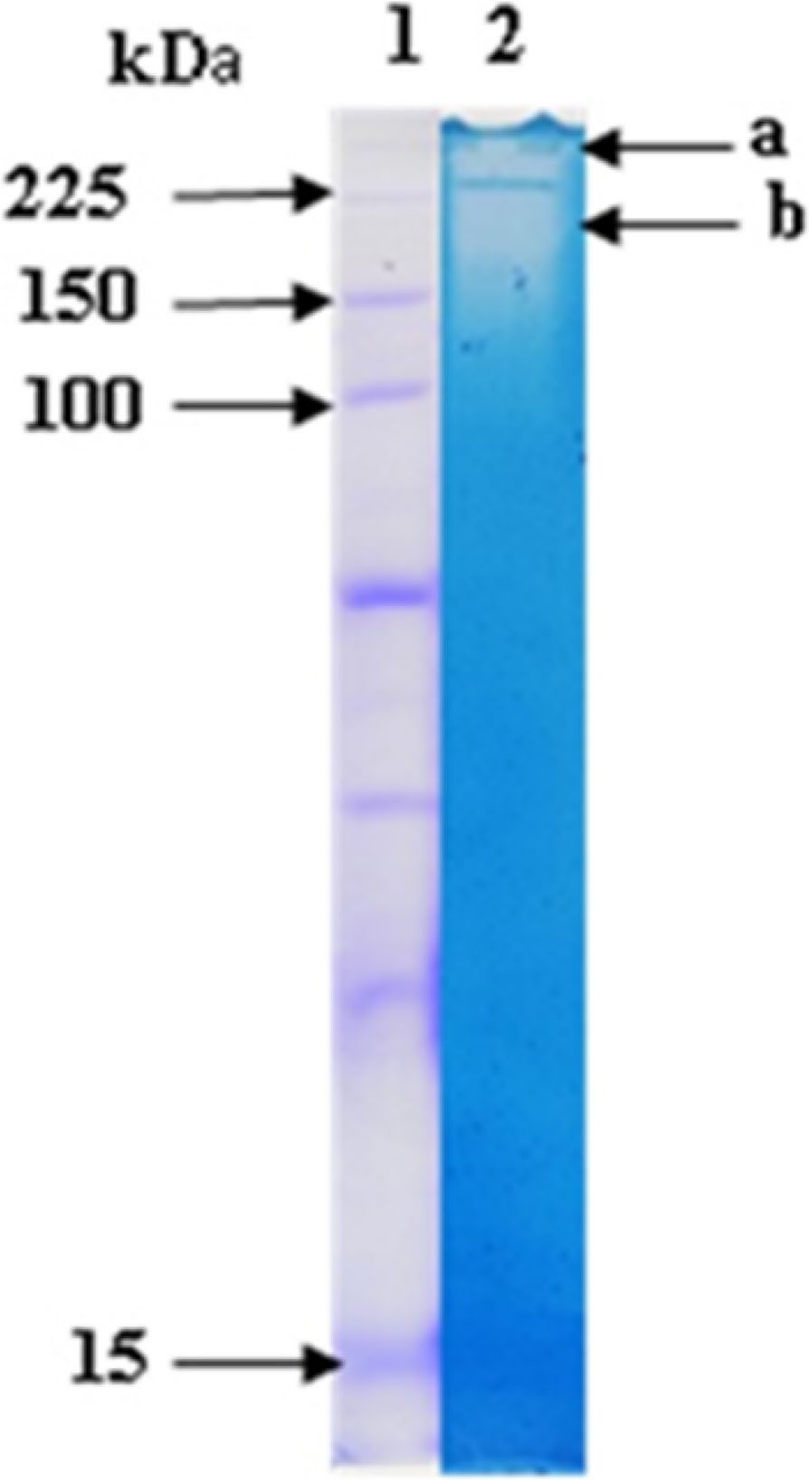
Zymography analysis of the protease activity from *Halobacillus* sp. HAL1. Lane **1**: protein markers; lane **2**: partially purified protease, a, and b represent the two casein degrading activities

The partially purified protease was analyzed by zymography. Two clear bands showed casein degradation activities, at approximately 250 and 190 KDa (Fig. 11).

### Effect of salts on protease activity

**Fig. 12:**
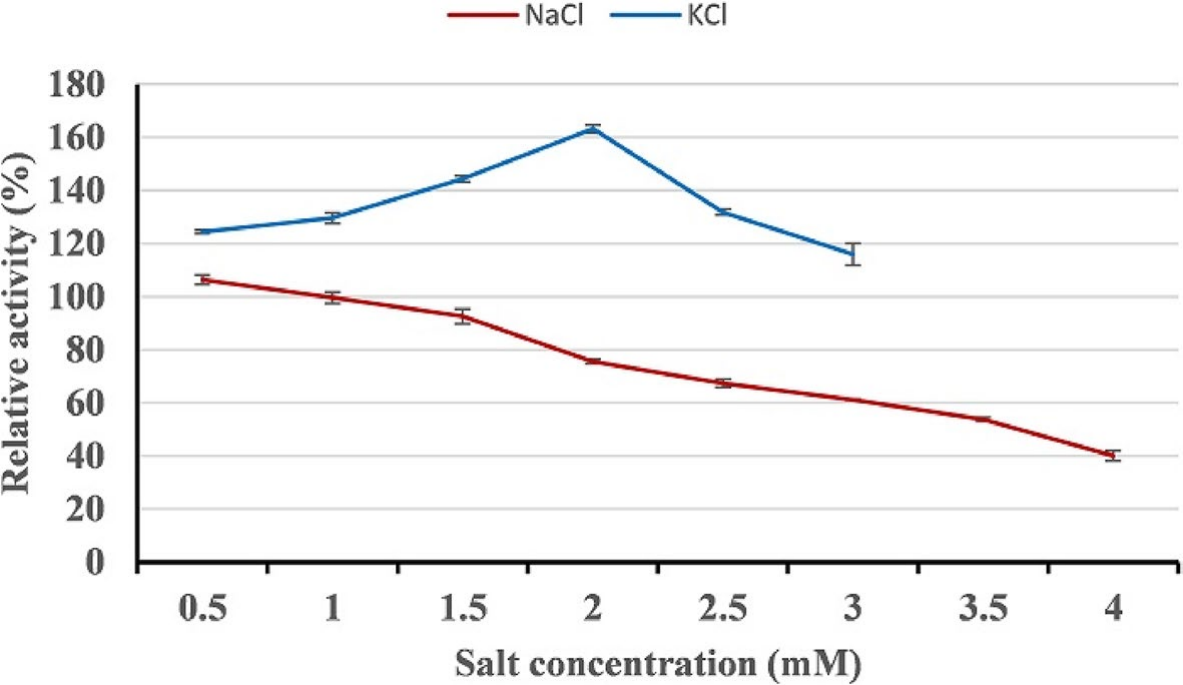
Effect of salts on the activity of HAL1 protease. The effect of salts was determined by incubating the enzyme with different concentrations of NaCl (0–4 M) and KCl (0–3 M) for 1 h and the enzyme activity was measured under standard assay conditions. The activity of the enzyme without NaCl and KCl was assumed to be 100%. The error bars show the standard deviation of three replicates

The effect of salts on HAL1 protease activity was studied using increasing NaCl (0 to 4 M) and KCl (0 to 3 M) concentrations. As shown in Fig. 12, the maximum relative activity of the enzyme was obtained at 0.5 M NaCl (106.5 ± 2.5). Beyond 0.5 M NaCl, enzyme activity decreased progressively, and only 40% of its relative activity remained at 4 M NaCl. However, the presence of up to 3 M KCl enhanced the enzyme activity, with maximum relative activity at 2 M KCl (163.3 ± 1.5).

### Effect of temperature on protease activity

**Fig. 13:**
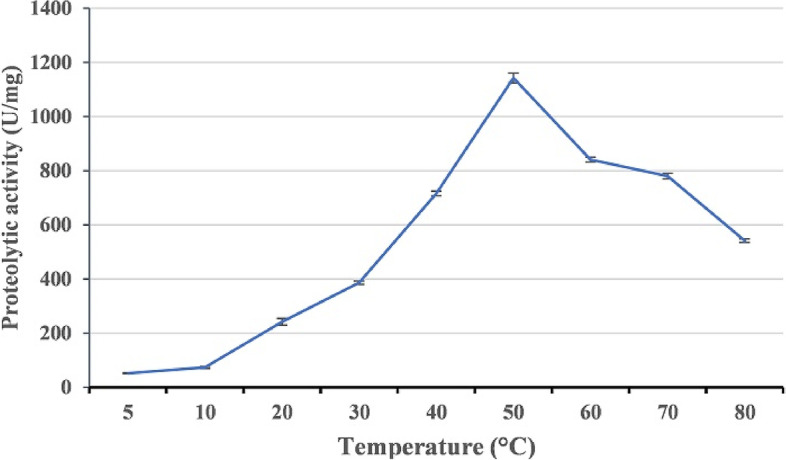
Effect of temperature on the activity of HAL1 protease. The effect of temperature was evaluated by assaying protease activity at different temperatures between 5 and 80 °C. The error bars show the standard deviation of three replicates

The effect of different temperatures (5–80 °C) on enzyme activity was investigated. According to our findings, the activity of the enzyme peaked at 50 °C and remained active up to 80 °C (Fig. 13).

### Effect of pH on protease activity

**Fig. 14:**
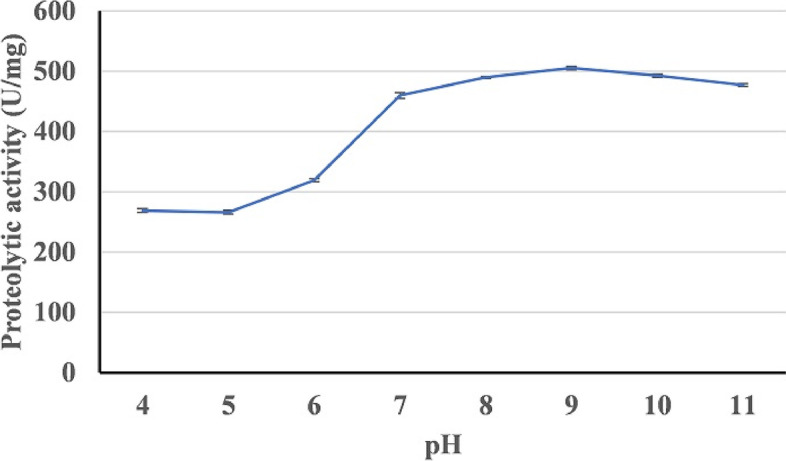
Effect of pH on the activity of HAL1 protease. The effect of pH was evaluated by assaying protease activity at different pH between 4 and 11. The error bars show the standard deviation of three replicates

The effect of pH on HAL1 protease was studied at different pH (4-11) at 50 °C using casein as substrate. The enzyme was significantly active between pH 7 and 11 with optimum activity (508.1 U/mg protein) at pH 9 (Fig. 14). The enzyme showed about 2.6 and 5.9% activity reduction at pH 10 and 11, respectively.

### Effect of organic solvents on protease activity

Table 2 summarizes the impact of some polar and non-polar organic solvents (− 0.24 ≤ log *P*_*o/w*_ ≤ 3.5), at 25% concentration, on HAL1 protease activity. The inclusion of hydrophobic solvents with a partition coefficient in the octanol/water two-phase (log *P*_*o/w*_) greater than 3 increased the activity of the enzyme. Organic solvents with log *P*_*o/w*_ ≤ 2.13, on the other hand, reduced the protease activity.

**Table 2:** Effect of organic solvents on HAL1 protease activity

Organic solvent (25%, v/v)	Log *P *_*o/w*_	Relative activity (%)
Control		100
Methanol	− 0.76	79.3 ± 0.6
Ethanol	− 0.24	75.3 ± 1
Acetone	− 0.24	78.6 ± 1.2
Hexane	3.5	112.6 ± 1.9
Benzene	2.13	87.9 ± 1.2
Propanol	0.25	27.8 ± 0.7
Butanol	0.8	16.8 ± 1.2
Xylene	3.1	162.3 ± 1.6

Data are means ± standard deviations (SD) for three replicates

### Effect of metal ions and EDTA on the activity of HAL1 protease

**Fig. 15:**
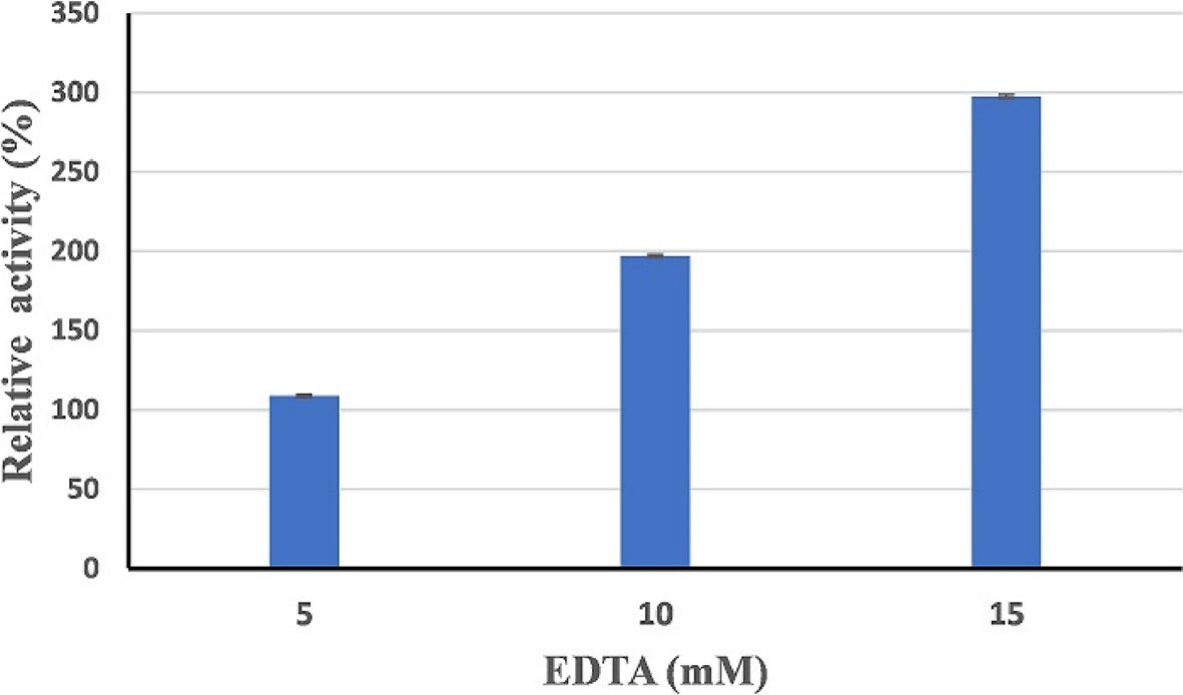
Effect of EDTA on the activity of HAL1 protease. The effect of EDTA concentration was evaluated by assaying protease activity at different concentrations (5, 10, and 15 mM). The error bars show the standard deviation of three replicates

Table 3 shows the effect of some divalent metal ions on the activity of HAL1 protease. According to our findings, the presence of Ca^2+^, Mg^2+^, and Mn^2+^ increased the activity of the enzyme. The presence of 10 mM Mn^2+^ ions resulted in the greatest increase in enzyme activity (1165.8% ± 4.7). Pb^2+^ ions had a minor inhibitory effect at 5 and 10 mM, while Zn^2+^ had the strongest inhibitory effect at 10 mM. The effect of different EDTA concentration (5, 10, and 15 mM) on HAL1 protease activity was studied. The enzyme activity was increased with increasing EDTA concentration. The respective relative activities were 118.2, 213.6, and 315% (Fig. 15).

**Table 3:** Effect of organic solvents on HAL1 protease activity

Metal ion	Relative activity (%)	
	5 mM	10 mM
No Metal	100	100
Fe2 +	37.3 ± 0.6	35.5 ± 0.9
Cd2 +	45.1 ± 0.7	38 ± 0.9
Cu2 +	95.4 ± 0.3	56.5 ± 0.9
Ca2 +	111.7 ± 0.4	127.7 ± 1.4
Mg2 +	112.3 ± 0.8	120.6 ± 0.1
Pb^2+^	98.6 ± 0.2	92.5 ± 0.9
Zn^2+^	43.1 ± 0.6	31.9 ± 0.3
Mn^2+^	905.1 ± 4.1	1165.8 ± 4.7

Data are means ± standard deviations (SD) for three replicates

### Effect of surfactants and H_2_O_2_ on the activity of HAL1 protease

The effect of surfactants and H_2_O_2_ on the activity of HAL1 protease was studied, and it was found that incubation with Tween-80, SDS, and H_2_O_2_ (1, 5, and 10%) stimulated the activity to varying degrees, with H_2_O_2_ having the highest stimulation effect at 10% concentration (227.4% ± 1.0). The presence of 1 and 5% concentrations of Triton-X100 caused a minor change in enzyme activity. However, the enzyme activity decreased sharply to 55.2% at 10% concentration of Triton-X100 (Table 4).

**Table 4:** Effect of surfactants and H_2_O_2_ on the activity of HAL1 protease

Surfactant/oxidizing agent	Relative activity (%)		
	1%	5%	10%
Control	100	100	100
Tween-80	108.4 ± 0.4	108.7 ± 0.7	106.1 ± 1.4
Triton-X100	100.7 ± 0.8	99.5 ± 0.4	55.2 ± 0.8
SDS	108.6 ± 1.0	119.6 ± 0.4	115.9 ± 1.3
H_2_O_2_	148.0 ± 1.4	189.3 ± 0.7	227.4 ± 1.0

Data are means ± standard deviations (SD) for three replicates

## Discussion

We isolated six bacterial isolates from saline soil, and one isolate, coded as strain HAL1, was chosen for its ability to produce alkaline protease. The strain was identified as a species of the genus *Halobacillus* and designated as *Halobacillus* sp. HAL1 with the GenBank accession number OK001470. In addition, we carried out further investigations concerning optimization of production, partial purification, and characterization of the protease produced by the strain. Our findings revealed that the enzyme has a high molecular weight and is compatible with surfactants, EDTA, metal ions, and organic solvents, indicating that it is suitable for a variety of industrial applications.

Thalssohaline environments are hypersaline environments that originate from the sea and contain salts that have an ionic composition similar to seawater. However, the concentration of seawater (3.5% salinity, on average) due to solar evaporation causes serial precipitation of various salts including, calcium carbonate, sodium chloride, and the salts of Mg^+2^ and K^+^ ions. These habitats are either naturally occurring or man-made salterns. Sabkhas, also known as saline soil or evaporites, are good examples of the natural hypersaline environments [34]. Only halophilic and halotolerant microorganisms thrive in these environments, which have lower microbial diversity than seawater [35]. The extreme saline conditions in these environments favors the growth of microbes possessing unique adaptive characteristics that could be exploited in a variety of biotechnological applications, particularly hydrolytic enzymes [36]. In this study, we isolated six isolates of heterotrophic bacteria from saline soil and assessed their capacity to produce alkaline protease. One isolate, designated HAL1, had the highest alkaline protease activity and was chosen for further characterization and production of alkaline protease. The isolate is a Gram-positive rod that requires sodium for growth, and can tolerate up to 24% of NaCl with optimum growth achieved at 9% of NaCl and, therefore, was considered a moderately halophilic bacterium [37].

Phylogenetic analysis of the 16 s rDNA of the isolate confirmed the affiliation of the isolate to the genus *Halobacillus* and it was designated as *Halobacillus* sp. HAL1; the sequence was deposited in the GenBank under the accession number OK001470. Over the past decades, microorganisms inhabiting saline and hypersaline environments have been the subject of interest for the bioprospecting of valuable hydrolytic enzymes [38–40]. Several studies have reported the isolation of halotolerant and halophilic microbes producing potent hydrolases that are active under extreme conditions of salinity and pH, especially members of the genus *Halobacillus* [41–45].

The cost-effective production of enzymes represents a great challenge for the industrial application of enzymes. Therefore, the use of low-cost substrates is urgently needed to enhance the use of enzymes in various industries in an economical way [46]. The utilization of fish waste substrate, which provides an excellent nutrient source for microbial growth and enzyme production, could solve this issue [18]. In the present study, HAL1 produced higher protease activity when grown in media containing only fish waste substrate, and this could lower the cost of enzyme production.

The low yield of enzymes and other metabolites from extremophiles is one of the major obstacles in their industrial applications [47]. The effects of culture media composition and the culturing conditions such as aeration level, temperature, pH, and incubation time on the production of alkaline protease have been confirmed [48–50]. To obtain a high yield of alkaline protease, it is critical to optimize the composition of the production medium and the culturing conditions. The optimal culturing conditions for alkaline protease production by HAL1 were studied, and the results revealed that the strain produces the highest yield of alkaline protease when cultured in an artificial seawater based medium containing 10 g/L of SC waste powder and 30 g/L of NaCl, pH 8 and incubated at 25 °C under shaking conditions (150 rpm). The ability of HAL1 to grow and produce protease in a medium containing only SC waste substrate alone indicates that this substrate could promote the growth and protease production without the need for additional nutrients. The requirement for alkaline pH for optimal protease production suggests the alkaliphilic nature of the strain and the produced protease [22, 51]. In addition, the enhanced protease production under shaking conditions indicates the aerobic nature of strain HAL1 [52].

Microbial proteases have molecular masses ranging from 15 to 40 kDa [16, 53]. Thus, Karbalaei-Heidari et al. [54] identified an extracellular alkaline protease from the moderately halophilic bacterium *Halobacillus karajensis* with molecular weight of 36 kDa, whereas alkaline protease from *Halobacillus andaensis* is about 18 kDa [55] and from *Halobacillus* sp. CJ4 of 18 to 30 kDa [56]. Santos et al. [57] detected several proteases with molecular masses ranging from 30 to 80 kDa in *Halobacillus blutaparonensis*. However, Dorra et al. [58] identified a high molecular weight alkaline protease of about 250 kDa produced by *Bacillus halotolerans* strain CT2. The partially purified protease secreted by HAL1 showed two casein degradative activities with molecular masses of 190 and 250 kDa. To our knowledge, this is the first report on a high molecular weight protease from a *Halobacillus* species.

The enzymes of halophilic microorganisms are adapted to function in hypersaline environments and possess unique properties, including thermostability and pH tolerance. In addition, halophilic enzymes are resistant to denaturation and can catalyze in low water activity [59–62]. Among halophilic enzymes, proteases find a wide application in pharmaceuticals, leather tanning, food, and detergent industries due to its stability under harsh industrial conditions [36]. The effects of salts (NaCl and KCl) and pH on the activity of HAL1 protease were studied, and our results revealed that 0.5 M NaCl, 2 M KCl, and pH 9 are the optimum conditions for maximum activity of the enzyme. These results indicate the halo-alkaline nature of the enzyme and are similar to the optimal conditions for proteases from *Halobacillus* sp. CJ4 strains [56] and *Halobacillus*. *karajensis* MA-2 [54]. Because HAL1 protease showed excellent thermostability at wide range of temperatures, from 30 to 80 °C, with an optimum temperature of 50 °C, it was considered a thermostable enzyme. a Similar temperature optimum was reported for alkaline protease from *Halobacillus karajensis* MA-2 [54] and *Bacillus mojavensis* [12].

Besides thermal stability and activity at high pH, proteases that are stable in the presence of organic solvents, oxidizing agents, metal ions, and surfactants are attractive for industrial applications [63]. Protease-catalyzed reactions are often carried out in non-aqueous media, so proteases that are stable in the presence of organic solvents would be valuable for synthesis in such environments [64]. According to Laane et al. [65], the logarithm of the partition coefficient of the solvent between octanol and water (log *P*_*o/w*_) is the best parameter for relating the enzyme activity to the solvent nature. Thus, hydrophobic solvents (having high log *P*_*o/w*_ values) cause less inactivation of biocatalysts than solvents with lower log *P*_*o/w*_ values. The presence of hexane and xylene (log *P*_*o/w*_ greater than 3) induced the activity of HAL1 protease, whereas organic solvents having log *P*_*o/*_w less than 0 or equal to 2.1 deactivated the enzyme by about 20%. Butanol (log *P*_*o/w,*_ 0.8) deactivated HAL1 protease by 84%. Hydrophilic solvents destabilize enzymes by removing the water hydration shell of the enzyme which is essential for structure flexibility and catalytic activity [66], and this could account for the decrease in HAL1 protease activity in the presence of hydrophilic solvents.

The effect of some divalent metal ions on the activity of HAL1 protease was studied. Among the tested metal ions, the presence of Ca ^2+^, Mn ^2+^, and Mg ^2+^ (5 and 10 mM) enhanced the enzyme activity; the highest activation effect was observed in the presence of 10 mM Mn^2+^ ions (1165% relative activity). Similarly, Ca^2+^ and Mg^2+^ ions have previously been shown to activate protease enzyme [67–74]. Yu et al. [75] found that Mn^2+^ ions (10 mM) had a lower activation effect on alkaline protease from *Bacillus* sp. ZJ1502, compared with the effect on HAL1 protease (122 and 1165% relative activity; respectively). The significant enhancement of the enzyme caused by the addition of Mn^2+^ ions suggests that this metal ion facilitates the binding of the substrate to the active site of the enzyme [75]. On the other hand, Zn^2+^ and Fe^2+^ ions partially inhibited the enzyme; similar inhibition effects have been reported for alkaline protease from *Bacillus* sp. ZJ1502 [75] and *Bacillus halotolerans* strain CT2 [58]. Interestingly, HAL1 protease was stable in the presence of Pb^2+^ ions (5 and 10 mM) but lost about 70% of its activity in the presence of Cd^2+^ ions. Some metal ions may inhibit protease activity by binding to specific amino acids that are important for catalytic function or by affecting the charge distribution of the enzyme molecules [76]. The activity of HAL1 protease was increased in the presence of EDTA (5, 10, and 15 mM), and the relative activities were 118.2, 213.6, and 315%, respectively. The significant increase of enzyme activity in the presence of EDTA is a novel finding and suggests the enzyme is not a metalloprotease enzyme [77] and is suitable for the detergent industry, particularly because chelating agents such as EDTA are commonly used in detergent formulation [68, 71].

Surfactants and oxidizing agents are commonly used in the formulation of modern detergents. Therefore, alkaline proteases that are stable in the presence of oxidizing agents and surfactants are crucial in the detergent industry [68]. Incorporation of Tween 80, SDS, or H_2_O_2_ (up to 10%) and Triton-X 100 (up to 5%) into the reaction mixture enhanced the activity of HAL1 protease; the relative activity in the presence of 10% H_2_O_2_ was 227.1%. Similar activation effect of Tween 80 and Triton- × 100 have been reported previously for alkaline protease from *Bacillus invictae* [15], while a higher activation effect was observed for serine alkaline protease from *Bacillus safensis* strain RH12 [78] and crude protease from *Bacillus cereus* SV1 [79]. Lower activation effect of oxidizing agents for other alkaline proteases have been reported [68, 75, 79–83]. However, protease from *Aeribacillus pallidus* C10 showed weak stability in the presence of H_2_O_2_ [84]. In comparison to all of these proteases, HAL1 protease showed a high level of oxidizing agents compatibility. In addition, stability of HAL1 protease was improved in the presence of up to 10% SDS; few studies have reported the stability of proteases in the presence of SDS [84].

## Conclusion

In the current study, the moderately halophilic bacterium, *Halobacillus* strain HAL1 was isolated from saline soil and used to produce an extracellular alkaline protease using fish waste substrate as the sole nutritional source. The enzyme secreted by HAL1 strain was found to be a novel high molecular weight alkaline protease with molecular masses of 190 and 250 kDa and to exhibit novel properties that make it suitable for detergent formulations such as alkaline pH and thermal stability, as well as high compatibility with metal ions, organic solvents, surfactants, EDTA, and H2O2. Further research is needed to fully understand its structure organization, as well as the possible industrial applications.

## Data Availability

All data generated or analyzed during this study are included in this published article.

## Abbreviations

CAGR: Compound annual growth rate
FPW: Fish processing waste
SC: *Scarus collana*
PCR: Polymerase chain reaction
DNA: Deoxyribonucleic acid
BLAST: Basic local alignment search tools
SDS: Sodium dodecyl sulphate
EDTA: Ethylenediaminetetraacetic acid
min: Minutes
h: Hours
